# Instability Resistance Training Decreases Motor Noise During Challenging Walking Tasks in Older Adults: A 10-Week Double-Blinded RCT

**DOI:** 10.3389/fnagi.2019.00032

**Published:** 2019-02-27

**Authors:** Nils Eckardt, Noah J. Rosenblatt

**Affiliations:** ^1^Department of Training and Movement Science, Institute for Sport and Sports Science, University of Kassel, Kassel, Germany; ^2^Department of Sport and Movement Science, Institute of Sport Science, Carl von Ossietzky University of Oldenburg, Oldenburg, Germany; ^3^Dr. William M. Scholl College of Podiatric Medicine’s Center for Lower Extremity Ambulatory Research (CLEAR), Rosalind Franklin University of Medicine and Science, North Chicago, IL, United States

**Keywords:** irregular surface, unstable resistance training, uncontrolled manifold, motor redundancy, elderly, gait, perturbation

## Abstract

Locomotor stability is challenged by internal perturbations, e.g., motor noise, and external perturbations, e.g., changes in surface compliance. One means to compensate for such perturbations is to employ motor synergies, defined here as co-variation among a set of elements that acts to stabilize, or provide similar trial-to-trial (or step-to-step) output, even in the presence of small variations in initial conditions. Whereas evidence exists that synergies related to the upper extremities can be trained, the extent to which lower limb synergies, such as those which may be needed to successfully locomote in complex environments, remains unknown. The purpose of this study was to evaluate if resistance training (RT) in unstable environments could promote coordination patterns associated with stronger synergies during gait. Sixty-eight participants between the age of 65 and 80 were randomly assigned to one of three different RT modalities: stable whole-limb machine-based RT (S-MRT), instability free-weight RT (I-FRT), and stable machine-based adductor/abductor RT (S-MRT_HIP_). Before and after RT, participants walked across an even lab floor and a more challenging uneven surface with and without holding a weighted bag. The uncontrolled manifold control analysis (UCM) was used to calculate the synergy index (i.e., strength of the kinematic synergy) related to stabilization of our performance variable, the mediolateral trajectory of the swing foot, under each condition. Regardless of RT group, there was no effect of RT on the synergy index when walking across the even lab floor. However, the synergy index during the two uneven surface conditions was stronger after I-FRT but was not affected by the other RT modalities. The stronger synergy index for the I-FRT group was due to improved coordination as quantified by an overall increase in variability in elemental variable space but a decrease in the variability that negatively affects performance. The unstable environment offered by I-FRT allows for exploration of motor solutions in a manner that appears to transfer to challenging locomotor tasks. Introducing tasks that promote, rather than limit, exploration of motor solutions seems to be a valuable exercise modality to strengthen kinematic synergies that cannot be achieved with traditional strengthening paradigms (e.g., S-MRT).

**Clinical Trial Registration:** www.ClinicalTrials.gov, identifier NCT03017365.

## Introduction

Falls are a leading cause of injuries and mortality in older adults and the risk of falling increases with age (Rubenstein, 2006). Many falls in community-dwelling older adults occur during locomotion, particularly when postural stability is challenged by perturbations like slips and trips (Berg et al., 1997). Locomotor stability is generally realized through accurate positioning of the swing-foot relative to the center of mass (CoM) (Bruijn and van Dieën, 2018). In the frontal plane this requires active control by the central nervous system (Kuo, 2002; Bruijn and van Dieën, 2018) realized through activation of the swing limb hip musculature in response to states of the stance limb (Bruijn and van Dieën, 2018). However, increased neuromotor noise and associated motor variability (Kang and Dingwell, 2009) may negatively affects control of mediolateral (ML) foot placement and increases variability in ML placement of the foot (Bruijn and van Dieën, 2018), which may increase fall risk (Brach et al., 2005). Nonetheless, if an individual can compensate for increased variability, by channeling it into a subspace that does not affect performance, then the high variability would not be hazardous. The uncontrolled manifold (UCM) analysis provides a means to quantify the extent to which motor variability may or may not “be hazardous” (Latash et al., 2007).

The UCM analysis quantifies the extent to which all available degrees of freedom (DoF) that contribute to a task-relevant performance variable co-vary so as to stabilize (limit trial-to-trial variation in) that performance variable (Scholz and Schöner, 1999; Latash et al., 2007). The analysis decomposes variability in a set of elemental variables into two components: “good” variance that has no effect on the performance variable and “bad” variance that results into deviations of the performance variable. A positive synergy index, which quantifies the relative amount of “good” variance compared to “bad” variance, implies that the performance variable is stabilized by a synergy (Scholz and Schöner, 1999; Latash et al., 2007). Such stabilization allows secondary tasks that rely on the same set of elemental variables to be performed without affecting the primary task. It is generally thought that the effects of aging on motor coordination manifest as low amounts of “good variance” (Kapur et al., 2010). However, in response to challenging locomotor conditions such as uneven surface walking, healthy community-dwelling older adults are able to counteract perturbation-related increases in “bad” variability by channeling elemental variability into “good” variability (Eckardt and Rosenblatt, 2018). Similar findings have been reported for upper extremity tasks (Kapur et al., 2010). Thus, the aging human CNS possess the ability to harness motor flexibility, i.e., to increase the synergy index by increasing “good” variance through exploration of motor solution space. Given its’ importance, there is a need to understand whether this ability is trainable and if so, what exercises are optimal to train this ability.

With regard to the upper extremity, it has been demonstrated that non-repetitive tasks performed under conditions of manipulated stability can help promote large amounts of “good” variance (Shim et al., 2008; Wu and Latash, 2014). Similarly it has been suggested that exercise interventions which introduce tasks that promote, rather than limit, exploration of motor solutions may be particularly appropriate for promoting motor flexibility and the coordination patterns utilized to ambulate in complex environments (Rosenblatt et al., 2014). In turn, such exercises may help to reduce risk of falling and their introduction into fall prevention interventions would represents a considerable departure from traditional exercises, such as resistance training (RT) that targets muscle strength and power to achieve this goal (Benichou and Lord, 2016). Indeed, machine-based resistance training (S-MRT) does not seem suited to promote exploration of motor solutions due to restricted movements during exercise execution; a combination of balance and resistance training, i.e., “instability free-weight resistance training” (I-FRT), may be better suited to do this.

Instability free-weight RT is an exercise modality which involves tasks that specifically promote exploration of motor solutions by having participants engage in RT training while standing on destabilizing surfaces. The inherent instability during execution of the I-FRT results in greater overall muscle activation of the lower limbs due to the constant need for postural readjustment (Lawrence and Carlson, 2015). Indeed, the greater demands of I-FRT may explain why in our recent 10-week RCT I-FRT elicited similar increases in balance, power, and strength in older adults compared to S-MRT despite using half the training load (Eckardt, 2016). We hypothesized that inter-and intramuscular coordination may be the driving reason for increases in the respective outcomes. Nonetheless, strength, power, and balance are measures of performance that do not directly address changes in coordination such as those quantified by synergies within the UCM analysis. There is evidence, albeit limited, that RT can improve synergies; one study has evaluated the effects of finger RT on finger synergies, independence, force control and adaptations in multi-finger coordination (Shim et al., 2008). If RT does impact synergies, then RT focusing on the hip could be particularly beneficial with regards to improving kinematic synergies related to the mediolateral trajectory of the swing-foot during gait. Indeed, swing limb hip abductors activity is critical in modulating foot placement and is predicted by the relationship between the CoM and the stance limb (Rankin et al., 2014; Roden-Reynolds et al., 2015). On the other hand, strengthening may not significantly affect swing limb mechanics, which in part contribute to kinematic synergies related to foot placement. For example, increasing strength by 26% (i.e., control condition relative to a weaker nerve block condition) does not affect swing limb kinematics (Pohl et al., 2015). In fact, it is entirely possible that weaker older adults employ stronger synergies to compensate for weakness, as has been argued to occur during sit-to-stand tasks (Greve et al., 2013) such that hip strengthening could lead to a reduction in synergies. Thus, the extent to which RT, and particularly hip-specific RT, can impact motor coordination during locomotion remains unclear.

The purpose of the current study was to quantify how different RT modalities affect kinematic synergies related to the mediolateral trajectory of the swing-foot during normal and perturbed gait (walking across an uneven surface with and without additional asymmetric loading that promote additional imbalance). In addition to evaluating the effect of I-FRT and standard S-MRT on kinematic synergies, we also evaluated a highly specific adductor/abductor resistance training (S-MRT_HIP_) to better understand the extent to which hip strength affects kinematic synergies related to foot placement. We hypothesized first that kinematic synergies (i.e., synergy index) would stay invariant across groups from pre- to post testing during normal walking, given that normal walking is a habitual task. Second, we hypothesized that only the I-FRT group would increase the kinematic synergy index during perturbed gait (in absence of prior literature, we assumed the null for S-MRT_HIP_). Third, we hypothesized that in I-FRT the increase of the kinematic synergy index would result from an increase in “good” variance and a decrease in “bad” variance due to improved co-variation of lower-extremities based a previous study (Wu and Latash, 2014).

## Methods

### Study Design

We conducted a registered three-arm, double-blinded RCT (ClinicalTrials.gov: NCT03017365 on 01/04/2017) examining the effects of three (RT) protocols on kinematic synergies and strength, power, and balance in older adults. The assessors were blinded to the participants’ assignments. Participants were naïve to the study hypothesis. The trial was approved by the local ethics committee of the University of Kassel (E052016058) and was complied with the relevant ethical standards of the latest Declaration of Helsinki (WMA, October 2013). All participants provided written informed consent prior to enrollment.

### Participants

**FIGURE 1:**
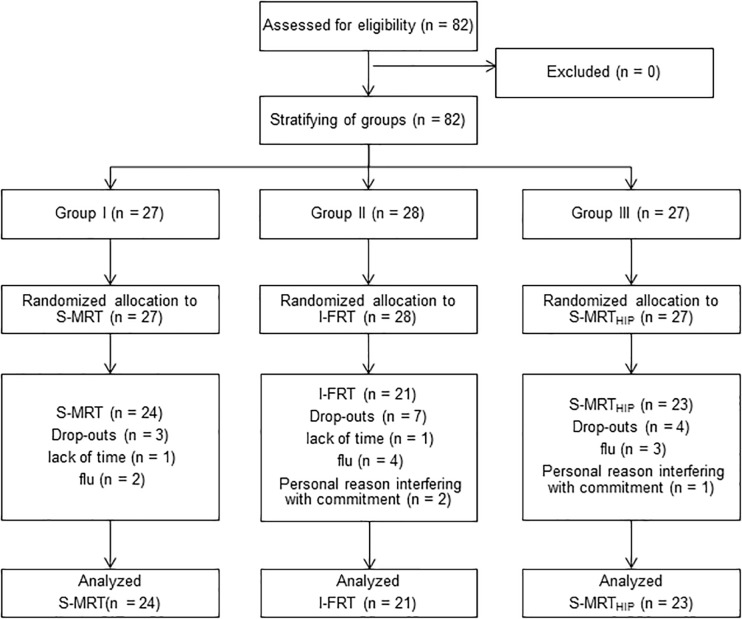
CONSORT diagram with participant flow. S-MRT, stable machine-based resistance training; I-FRT, instability free-weight resistance training; S-MRT_HIP_, stable machine-based adductor/abductor resistance training.

In total 82 participants between the age of 65 and 80 were recruited via public advertisement. The only inclusion criteria were the ability to walk independently without any gait aid. Participants were excluded based on pathological ratings of the Clock Drawing Test (CDT) (Nair et al., 2010), the Mini-Mental-State-Examination (MMSE, <24 points) (Lopez et al., 2005), the Falls Efficacy Scale – International (FES-I, >24 points) (Dias et al., 2006), the Geriatric Depression Scale (GDS, >9 points) (Parmelee and Katz, 1990), the Freiburg Questionnaire of Physical Activity (FQoPA, <1 h) (Frey and Berg, 2002) and the Frontal Assessment Battery (FAB-D, <13 points) (Benke et al., 2013). Ultimately, 68 participants successfully completed the trial. Figure 1 shows the CONSORT flow diagram and the number of participants in the treatment arms at each stage of the trial. Subject’ demographics and baseline descriptors of the who completed the 10-week trial are presented in Table 1.

**Table 1:** Subject characteristics and descriptive values.

	S-MRT (*n* = 24)		I-FRT (*n* = 21)		S-MRT_HIP_ (*n* = 23)		Baseline difference
Characteristics	*M*	*SD*	*M*	*SD*	*M*	*SD*	*p-*value
Age (years)	69.5	3.8	71.3	3.9	69.9	3.9	0.288
Body height (cm)	169	7	171	9	169	9	0.820
Body mass (kg)	73.8	12.4	76.9	15.7	76.6	13.6	0.691
Sex (f/m)	16/8		12/9		13/10		–
Physical activity (h/w)	11.9	8.6	9.4	9.2	12.2	7.2	0.215
MMSE	27.8	1.8	27.9	1.6	28.0	1.6	0.914
CDT	all participants were classified as non-pathological						
GDS	1.1	1.6	0.9	1.0	1.0	1.4	0.957
FAB_D	15.3	2.0	15.1	2.1	15.7	2.2	0.521


*S-MRT, stable machine based resistance training; I-FRT, instability free-weight resistance training; S-MRT_*HIP*_, stable machine based adductor/abductor resistance training; M, mean; SD, standard deviation; f, female; m, male; MMSE, Mini Mental State Examination; CDT, Clock Drawing Test; GDS, Geriatric Depression Scale; FAB_D, Frontal Assessment Battery, German Version.*

### Randomization

Participants were stratified (1:1:1) into one of three groups according to age and sex. An uninvolved researcher then randomly assigned the groups to one of three training modalities: Machine-based stable resistance training (S-MRT), free-weight instability resistance training (I-FRT), or machine-based adductor/abductor resistance training (S-MRT_HIP_). The randomization sequence was generated using www.randomizer.org and was concealed until groups were stratified.

### Assessment

Data was collected in the biomechanics laboratory of the University of Kassel, Germany.

#### Kinematic Data Collection, Processing and Analysis

**FIGURE 2:**
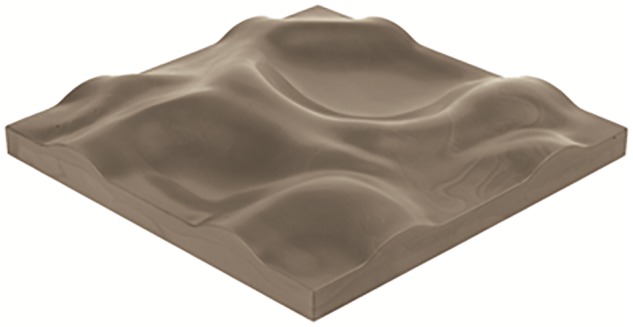
Artificial uneven surface. The terrasensa^®^ foam panels consist of polyurethane with the following material properties: shore durometer hardness scale A (DIN 53505) = 50 ± 5; impact resilience (DIN 53512) *R* = 50.

Twenty-six 12.5 mm reflective markers were attached bilaterally to the legs with double-sided adhesive tape at prominent bony landmarks according the IOR lower-body marker-set (Leardini et al., 2007). A six-camera motion capture system (Oqus 3+, Qualisys AB, Gothenburg, Sweden) operating at 120 Hz was used to record marker trajectories. Participants then walked for 1 min back and forth through a capture volume of 5 m at a self-selected walking speed. The capture volume was preceded/proceeded by ∼2 m which allowed the participant to accelerate/decelerate before entering/exiting the capture volume. Participants completed three conditions: the even surface (ES) (control) condition where they walked across the lab floor; the uneven surface (US) condition where foam panels (terrasensa^®^ classic; Huebner, Kassel, Germany; see Figure 2) were placed on the floor to create an uneven surface; and the “imbalanced shopping bag” (ISB) condition where they walked across the US carrying a simulated shopping bag – i.e., a tube, lanced with a chain with ends weights attached (5% of the body weight) – in the dominant hand (US_ISB_). The last condition was intended to present an additional challenge to balance above the US condition alone. All data were processed using Visual3D (C-Motion, Germantown, MD, United States). Raw kinematic marker trajectories were interpolated and smoothed with a fourth-order zero-lag Butterworth low-pass filter with a cut-off frequency of 6 Hz. The UCM-analysis was then performed on the processed data using a custom written R-code (R Foundation for Statistical Computing, Vienna, Austria). The custom code is available upon request.

#### Primary Measures: Uncontrolled Manifold Analysis

The UCM approach has been described in detail elsewhere (Scholz and Schöner, 1999; Latash et al., 2007) as has its application to stabilizing the swing limb trajectory during gait (Rosenblatt et al., 2015; Eckardt and Rosenblatt, 2018). Briefly, motion capture data was normalized to 0–100% corresponding to left toe off to heel strike. A geometric model was used to express the position of the swing limb at each percent of swing as a function of seven lower limb segment angles. For each step and at each percent of swing, the deviation between each angle and it’s across-step average was calculated. A deviation vector was then projected onto a 6-DOF space that did not affect the swing limb position and a 1 DOF space that did, based on the Jacobian of the geometric model. The across-step average length of the projected vectors defined “good” and “bad” variance, respectively, at every percent of swing from which a synergy index was expressed. Consistent with prior studies (Krishnan et al., 2013; Rosenblatt et al., 2015; Eckardt and Rosenblatt, 2018), the variance components and synergy index were averaged across the swing phase for further analysis. The primary outcome from the analysis is the synergy index, which was z-transformed prior to statistical testing (ΔV_Z_). The index was calculated as the difference between the “good” variance per DOF (V_UCM_) and “bad” variance per DOF (V_ORT_) relative to the total kinematic variance per all DOFs (V_TOT_) such that changes in ΔV_Z_ can reflect multiple strategies (Wu and Latash, 2014). Therefore, in addition to ΔV_Z_, we also report V_UCM_, V_ORT_, and V_TOT_. A more detailed description of the UCM method can be found in Supplementary Material SII.

#### Secondary Measures

##### Balance assessment

We tested proactive balance using the timed-up-and-go test (TUG) (Podsiadlo and Richardson, 1991) and the multidirectional reach test (MDRT) (Newton, 2001). For the TUG, participants were asked to rise from a chair and walk three meters at their habitual walking speed, turn around a cone return to the chair and then sit down. Time was recorded to the nearest 0.01 s using a stopwatch that started on the command “ready-set-go” and stopped as soon as the participants sat down. The MDRT measures the maximal distance participants could reach forward, backward, left, and right while standing without taking a step. Maximal reach distance (cm) was recorded. We added the left/right conditions and calculated the mean mediolateral distance for further analysis. Participants had one practice trial for every test. Two test trials were carried out and the mean was entered into statistical analysis.

##### Strength and power assessment

Maximal isometric leg extension strength was examined with the isometric mid-thigh pull test (IMTP) (McMaster et al., 2014). Data was measured with a force plate (Model 9281B, Kistler Instrument AG, Winterthur, Switzerland), operating at 1200 Hz and recorded with QTM (Qualisys AB, Gothenburg, Sweden). Participants stood upright in a squatting position on a solid, elevated metal platform, bridging over the force plate to avoid contact. Cable length was individualized to guarantee a constant knee angle of approximately 135°. Participants were then asked to pull upward on a handle connected to the force plate, starting initially with a moderate intensity and slowly increase the intensity to maximum exertion while keeping the upper body extended and upright. To ensure upright posture, an assessor put her hand on the participants’ back while pulling. The IMTP shows high within- and between-session reliability (ICC ≥0.87) (Moeskops et al., 2018).

Bilateral isometric strength of hip adduction and abduction, as well as the knee extensor, was measured with a hand-held dynamometer (Lafayette Instrument Company, Lafayette, IN, United States) (Arnold et al., 2010). To measure hip adduction and abduction, participants were positioned sideways on a therapy bench. The hand-held dynamometer was placed above the malleolus of the lower (adduction) or upper leg (abduction) respectively, as previously described (Arnold et al., 2010). Participants were asked to adduct and abduct their respective leg. For knee extension strength, participants sat on the therapy bench and were asked to try to extend their leg. The assessor placed the hand-held dynamometer at the lower leg just proximal to the ankle. We recorded two maximum effort isometric contractions for 3–5 s with each muscle group. Interclass correlation coefficients (ICCs) are generally high for hand-held dynamometry (ICCs 0.95–0.99) (Arnold et al., 2010). For all isometric strength testing, we provided one practice trial and then averaged the next two trials. Measures were taken on both limbs and then averaged across limbs prior to statistical analysis. To limit the effects of fatigue we allowed recovery periods (>1 min) between trials.

To assess lower extremity muscle power, we administered the Five Times Sit-to-Stand-Test (STS) (Tiedemann et al., 2008). Participants were instructed to stand up and sit down five times as quickly as possible, without using their arms. They were advised to fold their arms across the upper body. Time was measured by a stopwatch to the nearest 0.01 s. After the countdown “ready-set-go,” testing time was started and stopped when participants sat down for the fifth time.

##### Questionnaires

Global cognitive function was assessed using the MMSE, a screening tool for mild cognitive impairment (Lopez et al., 2005). The FAB-D consists of six neuropsychological tasks, evaluating cognitive and behavioral frontal lobe functions (Dubois et al., 2000). Physical activity was assessed using the FQPA (Frey and Berg, 2002). Concern about falling was evaluated using FES-I (Dias et al., 2006). The FES-I was the only questionnaire applied pre- and post-testing. All other tests were used for screening purposes and/or to describe the population.

### Exercise Intervention

The exercise intervention took place between January and April. Training was supervised by two trained instructors providing a participant to instructor ratio of 5:1. All intervention groups trained for 10 weeks, twice per week on non-consecutive days for 60 min per day. We began with a 1-week introductory phase and three training blocks lasting 3 weeks each. Training intensity was progressively and individually increased by modulating load and sets for all groups and the level of instability for the I-FRT group (see Table 2). After week one, four, and seven the training load (weight) was increased following one repetition maximum (1-RM) testing using the prediction equation provided by Epley (Reynolds et al., 2006) for each major exercise. The 1-RM was performed under stable conditions for every group.

**Table 2:** Detailed intervention program for all groups and phases.

	Intro-phase (1 week)	Block I (3 weeks)	Block II (3 weeks)	Block III (3 weeks)
	∼2 × 12 reps (with low weights, 2–3 min rest between sets and 5 min between exercises)	3 × 15 reps (50% of the 1-RM, 2–3 min rest between sets and 5 min between exercises)	3–4 × 15 reps (60% of the 1-RM, 2–3 min rest between sets and 5 min between exercises)	4 × 15 reps (60% of the 1-RM, 2–3 min rest between sets and 5 min between exercises)
**S-MRT**				
Cross-Trainer	10 min	10 min	10 min	10 min
Smith-Machine	150°knee flex/ext angle	120°knee flex/ext angle	100°knee flex/ext angle	100°knee flex/ext angle
Leg-Press	90°knee flex/ext angle	90°knee flex/ext angle	90°knee flex/ext angle	90°knee flex/ext angle
Core Exercise	Bridge exercise (2 × 15 reps)	Bridge exercise (3 × 20 reps)	Crunches (4 × 20 reps)	Air Bike Crunches (4 × 20 reps)
Walking with dumbbells	2 min without dumbbells	3 min with 5% of bw	4 min with 10% of bw	5 min with 15% of bw
**I-FRT**				
Cross-Trainer	10 min	10 min	10 min	10 min
Squats	150°knee flex/ext angle on AIREX^®^ coordination rocker board round	120°knee flex/ext angle on Thera-Band^®^ balance pads placed on AIREX^®^ coordination rocker board angled	100°knee flex/ext angle on AIREX^®^ balance pad placed on AIREX^®^ coordination rocker board angled	100°knee flex/ext angle on BOSU^®^ ball or Variosensa board
Front lunges	Thera-Band^®^ Balance Pads (front foot)	AIREX^®^ coordination rocker board round (front foot) and Thera-Band^®^ Balance Pads (rear foot)	AIREX^®^ balance pad (front foot) and Thera-Band^®^ Balance Pads (rear foot)	AIREX^®^ balance pad (front foot) and AIREX^®^ balance spinner soft (rear foot)
Core Exercise (Bridge Exercise)	No additional device (2 × 15 reps)	TOGU^®^ DYNAIR^®^ (under feet) (3 × 20 reps)	TOGU^®^ DYNAIR^®^ (under shoulder) and BOSU (under feet) (4 × 20 reps)	Swiss ball (under feet) (4 × 20 reps)
Walking with dumbbells	2 min without dumbbells on terrasensa^®^ flats	3 min with 5% of bw on terrasensa^®^ flats	4 min with 10% of bw on terrasensa^®^ classics	5 min with 15% of bw on terrasensa^®^
**S-MRT_HIP_**				
Cross-Trainer	10 min	10 min	10 min	10 min
Adductor	Habituation	Full ROM	Full ROM	Full ROM
Abductor	Habituation	Full ROM	Full ROM	Full ROM
Adductor Thera-Band^®^	Habituation	Full ROM	Full ROM	Full ROM
Abductor Thera-Band^®^	Habituation	Full ROM	Full ROM	Full ROM
Core Exercise	Side plank on knees (2 × 15 reps)	Side crunches (3 × 20 reps)	Standing oblique crunch (4 × 20 reps)	Russian sitting twist with dumbbell 5% bw, (4 × 20 reps)
treadmill walking on robowalk^®^	2 min habituation	3 min with ML pull above knee joint with 5% of bw	4 min with ML pull at ankles with 5% of bw	5 min with ML pull above knee joint and at ankles with 5% of bw, respectively


*S-MRT, stable machine-based resistance training; I-FRT, instability free-weight resistance training; S-MRT_*HIP*_, machine-based adductor/abductor training. bw, body weight; 1-RM, one repetition maximum; BOSU, BOth Sides Utilized; ROM, Range of Motion; ML, mediolateral.*

#### S-MRT

The main exercises of this group were squats at the Smith machine, placing the barbell at the hip instead of putting it on the participant’s shoulders, and the leg-press. Secondary exercise were core exercises and walking with weights across an even surface.

#### I-FRT

This group also performed squats, but instead of using the Smith machine, they exercised using instability devices (i.e., foam pads and BoSU balls) and dumbbells. The second main exercise was the front lunge on instability devices. Secondary exercises were core routines, incorporating instability devices, and walking across an uneven surface (terrasensa^®^ classic; Huebner, Kassel, Germany) carrying dumbbells.

#### S-MRT_HIP_

The main exercises for this group were the thigh/hip adductor- and abductor resistance machine. As secondary exercises, participants performed additional adduction and abduction exercises using elastic rubber straps. The resistance of the rubber straps was incrementally increased every block (changed by one color). Furthermore, lateral core exercises were introduced. In addition, this group walked across a special motorized treadmill (robowalk^®^, h/p/cosmos, Nußdorf, Germany), which applied a lateral pull via elastic straps at the ankle and/or knee while walking.

Detailed description of the training programs and changes in intensities and degrees of instabilities can be found in Table 2.

#### Training Intensity

Training intensity was quantified as the combined load (weight) for the two main exercises during the last training phase as determined from the participants’ training sheets.

### Data Analysis

An *a priori* sample size calculations with G^∗^Power 3.1.9.2 showed that to detect an expected effect of Cohen’s *d* = 0.3 (Shim et al., 2008; pilot data) at α = 0.05 with 1-β = 0.90 using a repeated measures with a within-between and interaction design, a total sample size of at least *N* = 15 per group was required. Normality of the data was checked by visual inspection and tested with the Kolmogorov–Smirnov test for each dependent variable per group, prior to the main analysis. Given that ANOVAs are quite robust against violations of distribution (Schmider et al., 2010), we would only employ non-parametrical alternatives in the event that a variable was non-normal for at least two groups. Baseline differences were tested between groups with a one-way ANOVA. Given that gait speed affects gait kinematics, we compared gait speed between pre- and post-testing for all three conditions with dependent two-sided *t*-tests. The effect of treatment was analyzed separately for each of the primary and secondary outcomes using 2 (*time*: pre-test, post-test) × 3 (*group*: S-MRT, I-FRT, S-MRT_HIP_) ANOVAs with repeated measures on *time* and between subject factor being *group*. In the case of a significant interaction (*p* ≤ 0.05), *post hoc* tests (dependent two-sided *t*-tests) were used to detect significant pre-post differences within each group. In addition, we investigated differences in the training load between groups using pre-planned independent two-sided *t*-tests. Ryan–Holm–Bonferroni corrected *p*-values for all *t-*tests are reported. Further, we employed Bayesian *t*-tests and calculated Bayes Factors (BF) to extend explanatory power of the inference *t*-tests results. We assume a default Cauchy prior width of 0.707. Table 3 summarizes the common interpretation of BF (Wetzels et al., 2011). To provide additional information, we also calculated the effect size as Cohen’s *d* for ANOVAs. Exploratory Software for Confidence Intervals was used to calculate Cohen’s *d_unb_* (an unbiased estimate of the population effect size *δ*), associated 95% confidence intervals and the *t*-tests (Cumming, 2012). Following Cohen (Cohen, 1988), *d*-values ≤ 0.49 indicate small effects, 0.50 ≤*d* ≤ 0.79 indicate medium effects, and *d* ≥ 0.80 indicate large effects. Alpha level was set at 5%. Bayesian *t*-tests were computed using JASP (Version 0.9.0.1). For all other tests we used IBM SPSS version 23.

**Table 3:** Evidence categories for Bayes factor.

Bayes factor	Interpretation
>100	Decisive evidence for H_A_
30–100	Very strong evidence for H_A_
10–30	Strong evidence for H_A_
3–10	Substantial evidence for H_A_
1–3	Anecdotal evidence for H_A_
1	No evidence
1/3–1	Anecdotal evidence for H_0_
1/10–1/3	Substantial evidence for H_0_
1/30–1/10	Strong evidence for H_0_
1/100–1/30	Very strong evidence for H_0_
<1/100	Decisive evidence for H_0_


*Adapted from Jeffreys (1961), cited in Wetzels et al. (2011). H0, Null-Hypothesis; HA, Alternative Hypothesis.*

## Results

**FIGURE 3:**
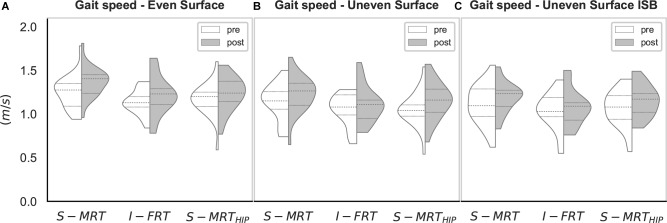
Violin-plot for gait speed. S-MRT, stable machine-based resistance training; I-FRT, instability free-weight resistance training; S-MRT_HIP_, stable machine-based adductor/abductor resistance training; ES, even surface; US, uneven surface; US_ISB_, uneven surface with imbalanced shopping bag; dashed line, Median; dotted line, upper/lower quartile. The width of the plots is scaled to data distribution.

**FIGURE 4:**
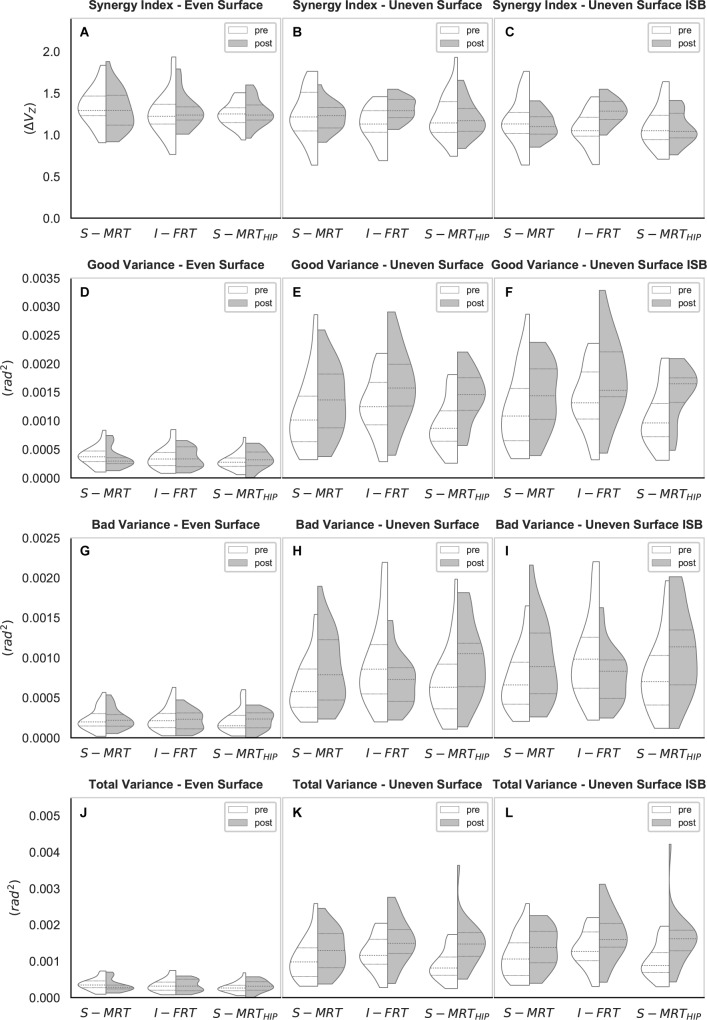
Violin-plots of the UCM gait analysis. **(A–C)** Synergy index across all three conditions; **(D–F)** “good” variance across all three conditions; **(G–I)** “bad” variance across all three conditions; **(J–L)** “total” variance across all three conditions. S-MRT, stable machine-based resistance training; I-FRT, instability free-weight resistance training; S-MRT_HIP_, stable machine-based adductor/abductor resistance training; ES, even surface; US, uneven surface; US_ISB_, uneven surface with imbalanced shopping bag; dashed line, median; dotted line, upper/lower quartile. The width of the plots is scaled to data distribution.

**FIGURE 5:**
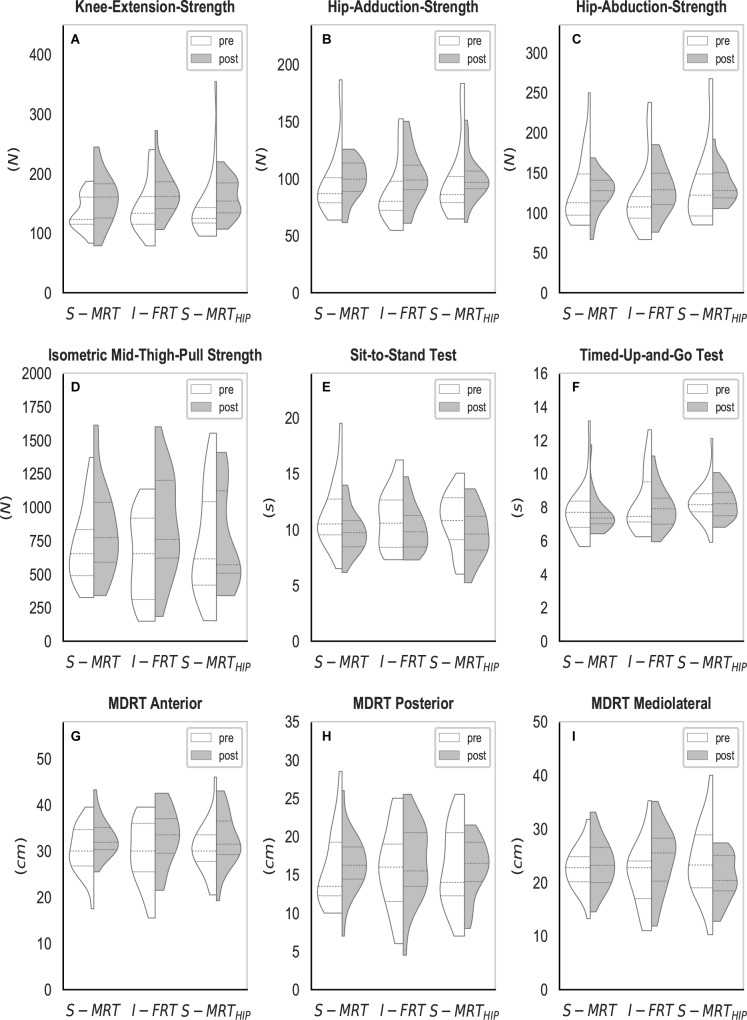
Violin-plots of the strength, power, and balance tests. **(A–C)** Isometric strength measured by a hand-held-dynamometer; **(D)** isometric leg strength; **(E)** lower-extremity power measured by the sit-to-stand test; **(F)** functional balance measured by the timed-up-and-go test; **(G–I)** balance measured by the multi-directional-reach test. S-MRT, stable machine-based resistance training; I-FRT, instability free-weight resistance training; S-MRT_HIP_, stable machine-based adductor/abductor resistance training; ES, even surface; US, uneven surface; US_ISB_, uneven surface with imbalanced shopping bag; dashed line, median; dotted line, upper/lower quartile. The width of the plots is scaled to data distribution.

The individual results are deposited as complete dataset in the Supplementary Material SI. The machine-based stable resistance training (S-MRT) group had an average attendance of 94%, 95% for the free-weight instability resistance training (I-FRT) group, and 95 % for the machine-based stable adductor/abductor resistance training (S-MRT_HIP_) group. Gait speed increased from pre- to post-testing for conditions (Figure 3): ES [*t*(66) = 3.74, *p* < 0.001, *d_unb_* = 0.45; 95%-CI (0.21, 0.71); BF_10_ = 61.67], US [*t*(66) = 3.89, *p* = 0.001, *d_unb_* = 0.41; 95%-CI (0.16, 0.67); BF_10_ = 21.84], and US_ISB_ [*t*(66) = 2.31, *p* = 0.024, *d_unb_* = 0.29; 95%-CI (0.04, 0.53); BF_10_ = 1.61]. Based on our *a priori* criteria for non-parametric testing, we were able to use parametric test for all variables. All outcomes are summarized in Figure 4, 5.

### Primary Measures: Uncontrolled Manifold

#### Kinematic Synergy Index

Regardless of group, ΔV_Z_ during the ES condition was not affected by RT (S-MRT: -4% change; I-FRT: 2% change; S-MRT_HIP_: 2% change). However, there was a *time x group* interaction for both challenging walking conditions (Table 4). In particular, ΔV_Z_ for I-FRT increased by 16% during US and 20% during US_ISB_ whereas there was no significant change in the kinematic synergy during either condition for both S-MRT and S-MRT_HIP_ (Table 5).

**Table 4:** ANOVA outcomes.

	Main effect time			Main effect group			Interaction time x group		
	*F*	*p*	*d*	*F*	*p*	*D*	*F*	*p*	*d*
**UCM ES**									
ΔV_ Z_	0.03	0.870	0.04	0.77	0.466	0.31	0.56	0.573	0.26
V_ UCM_	0.01	0.936	0.02	1.01	0.370	0.35	0.76	0.474	0.31
V_ ORT_	0.01	0.999	<0.01	0.17	0.842	0.14	0.12	0.887	0.13
V_ TOT_	0.01	0.986	<0.01	0.99	0.377	0.35	0.68	0.510	0.29
**UCM US**									
ΔV_Z_	2.33	0.132	0.38	0.07	0.929	0.09	4.11	**0.021**	0.71
V_UCM_	25.21	**<0**.**001**	1.24	1.62	0.205	0.45	0.98	0.381	0.35
V_ORT_	2.08	0.154	0.36	0.31	0.732	0.20	7.02	**0.002**	0.93
V_TOT_	26.76	**<0**.**001**	1.28	1.23	0.299	0.39	1.75	0.182	0.46
**UCM US_ISB_**									
ΔV_Z_	3.52	0.065	0.46	1.17	0.318	0.38	6.53	**0.003**	0.88
V_UCM_	20.16	<0.001	1.23	2.23	0.116	0.52	0.69	0.505	0.29
V_ORT_	2.52	0.117	0.39	0.33	0.722	0.20	7.04	**0.002**	0.93
V_TOT_	22.17	**<0**.**001**	1.17	1.61	0.208	0.44	1.36	0.265	0.41
**Balance**									
TUG	3.04	0.087	0.43	1.44	0.245	0.42	0.53	0.589	0.26
MDRT forward	14.33	**<0**.**001**	0.94	0.29	0.748	0.19	0.29	0.748	0.19
MDRT backward	0.69	0.411	0.20	0.05	0.953	0.06	0.11	0.896	0.11
MDRT ML	0.22	0.643	0.11	0.09	0.911	0.11	5.88	**0.004**	0.85
**Strength and**									
**power**
STS	26.34	**<0**.**001**	1.27	0.14	0.874	0.13	0.68	0.509	0.29
IMTP	58.64	**<0**.**001**	1.90	0.03	0.973	0.06	11.98	**0.001**	1.21
Hip adduction	2.57	0.117	0.39	0.29	0.752	0.19	0.91	0.407	0.33
Hip abduction	0.31	0.580	0.14	0.57	0.570	0.26	0.40	0.670	0.22
Knee extension	11.37	**<0**.**001**	0.84	0.63	0.535	0.28	0.16	0.852	0.14
**Questionaire**									
FES-I	12.36	**<0**.**001**	0.87	0.41	0.669	0.22	0.84	0.436	0.32
**Gait speed**									
ES	12.61	**<0.001**	0.89	4.61	0.013	0.76	0.32	0.730	0.20
US	10.13	**0.001**	0.80	2.03	0.014	0.51	0.50	0.608	0.25
US_ ISB_	4.97	**0.029**	0.56	1.14	0.327	0.38	0.30	0.744	0.19


*df_main_effect_: 1; *df_*interaction_effect*_*: 2, *df_*error*_*: 65; 2; S-MRT, stable machine-based resistance training; I-FRT, instability free-weight resistance training; S-MRT_*HIP*_, stable machine-based adductor/abductor resistance training; UCM, uncontrolled manifold approach; ΔV_*Z*_, synergy index; V_*UCM*_, “good” variability; V_*ORT*_, “bad” variability; V_*TOT*_, total variability; ES, even surface; US, uneven surface; US_*ISB*_, uneven surface with imbalanced shopping bag; TUG, timed up and go test; MDRT, multi directional reach test; ML, mediolateral; STS, sit-to-stand test; IMTP, isometric mid-thigh pull test; FES-I, falls efficacy scale. Bold values represent significant effects of the ANOVAs.*

**Table 5:** *T*-test pre-post comparisons.

		*t*	*p*	*d_unb_*	*95 CI d_unb_*	*BF*
UCM US						
ΔV_ Z_	S-MRT	-0.36	0.999	-0.08	–0.54, 0.38	0.23
	**I-FRT**	**4**.**68**	**<0**.**001**	**0**.**96**	**0.47, 1.51**	**200**.**58**
	S-MRT_HIP_	-0.21	0.999	-0.06	0.58, 0.47	0.22
V_ORT_	**S-MRT**	**2**.**61**	**0**.**036**	**0**.**55**	**0.11, 1.03**	**3**.**42**
	**I-FRT**	–**2**.**76**	**0**.**036**	–**0**.**54**	–**0.98,** -**0**.**12**	**8**.**50**
	**S-MRT_HIP_**	**2**.**07**	**0**.**050**	**0**.**56**	–**0.01, 1.15**	**1**.**31**
UCM US_ISB_						
ΔV_Z_	S-MRT	–0.61	0.999	–0.14	–0.61, 0.32	0.26
	**I-FRT**	**5**.**63**	**<0**.**001**	**1**.**15**	**0.63, 1.73**	**1421**.**66**
	S-MRT_HIP_	–0.16	0.999	–0.04	–0.57, 0.48	0.22
V_ORT_	**S-MRT**	**2**.**75**	**0**.**034**	**0**.**54**	**0.09, 1.01**	**3**.**13**
	**I-FRT**	–**2**.**80**	**0**.**033**	–**0**.**53**	**–0.96, -0.12**	**9**.**19**
	**S-MRT_HIP_**	**2**.**17**	**0**.**041**	**0**.**59**	**0.03, 1.19**	**1**.**54**
Balance						
MDRT ML	S-MRT	0.86	0.398	0.21	–0.29, 0.72	0.48
	**I-FRT**	**2**.**78**	**0**.**036**	**0**.**45**	**0.10, 0.82**	**8**.**91**
	S-MRT_HIP_	–2.08	0.098	–0.49	–0.99, 0.01	0.08
Strength						
IMTP	**S-MRT**	**3**.**85**	**<0**.**001**	**0**.**33**	**0.14, 0.55**	**83**.**57**
	**I-FRT**	**9**.**28**	**<0**.**001**	**0**.**65**	**0.42, 0.92**	**2.31^e+6^**
	S-MRT_HIP_	1.16	0.259	-0.09	–0.07, 0.25	0.68
Training						
Load
	**S-MRT vs. I-FRT**	**15.24**	**<0.001**	**4.34**	**3.34, 5.46**	**3.91^∗^10^16^**
	**S-MRT vs. S-MRT_HIP_**	**9.19**	**<0.001**	**2.64**	**1.88, 3.48**	**8.21^∗^10^08^**
	**I-FRT vs. S-MRT_HIP_**	**9.86**	**<0.001**	**2.72**	**1.98, 3.52**	**3.04^∗^10^10^**


*S-MRT, stable machine-based resistance training; I-FRT, instability free-weight resistance training; S-MRT_*HIP*_, stable machine-based adductor/abductor resistance training; M, mean; SD, standard deviation; UCM, uncontrolled manifold approach; ΔV_*Z*_, synergy index; V_*ORT*_, “bad” variability; US, uneven surface; US_*ISB*_, uneven surface with imbalanced shopping bag; TUG, timed up and go test; MDRT, multi directional reach test; ML, mediolateral; IMTP, isometric mid-thigh pull test. Bold values represent signifcant and meaningful effects of the t-tests.*

#### V_UCM_

Consistent with the fact that ΔV_Z_ during ES was not affected by any form of RT, we found no significant effect of time on “good” variance during the ES condition. In contrast, a main effect of *time* was observed for both unstable walking conditions. During the US condition we observed increases of 21% (S-MRT), 28% (I-FRT), and 50% (S-MRT_HIP_) for V_UCM_, with similar effects across groups; i.e., no significant interaction was present. Similar results were seen for the US_ISB_ condition; V_UCM_ for this condition increased by 19% following S-MRT, by 28% following I-FRT and by 43% following S-MRT_HIP_. There was no *time x group* interaction (Table 4).

#### V_ORT_

Regardless of group there was no significant effect of *time* on *V_ORT_* during the ES condition. However, there was a significant *time x group* interaction for both of the challenging conditions (Table 4). “Bad” variance decreased by 25% in the US condition following I-FRT and decreased by 24% in the US_ISB_ condition. In contrast to the I-FRT group, the other two groups significantly increased V_ORT_ by more than 35% during the US condition following training. Similar increases were found for these groups during the US_ISB_ condition; S-MRT increased “bad” variance by 35% and S-MRT_HIP_ by 41% (Table 5).

#### V_TOT_

Like V_UCM_ and V_ORT_, the total variance while walking across the even surface did not significantly change as a result of RT. However, there was a significant main effect of *time* on total variance during the two challenging conditions, with an average increase of 36% regardless of group; there was no significant *time x group* interaction for either challenging condition (Table 4).

### Secondary Measures

#### Balance Assessment

All groups reduced their TUG times by an average of 2%. However, there was no significant effect of *time* on TUG (Table 4).

The effects of RT on proactive balance, measured by the MDRT were consistent across groups; regardless of group, participants improved their forward reaching skills by an average of 8% whereas backward leaning was not significantly improved with RT. There was a significant *time x group* interaction for mediolateral proactive balance; with the S-MRT and I-FRT groups increasing side reaching by 4 and 14%, respectively, while S-MRT_HIP_ decreased their ability by 12% (Table 5). The *post hoc* tests revealed that the change for S-MRT and S-MRT_HIP_ was not significant whereas the effects were significant for I-FRT (Table 5).

#### Strength and Power Assessment

Regardless of group, lower extremity muscle power, measured using the Five Times Sit-to-Stand task increased by 10% in all groups. There was no significant *time x group* interaction (Table 4).

On average there was a 19% increase in isometric leg extension strength, but the effects varied by RT group; there was a significant *time x group* interaction. The *post hoc* tests revealed a significant improvement for S-MRT and I-FRT, and no significant effect observed for S-MRT_HIP_ (Table 5).

There was no effect of *time* on hip adduction and abduction strength. However, isometric knee extension strength did increase by 14% with time. Nonetheless, changes across groups were similar for all strength variables, thus we found no interaction effect *time x group* (Table 4).

#### Training Intensity

**FIGURE 6:**
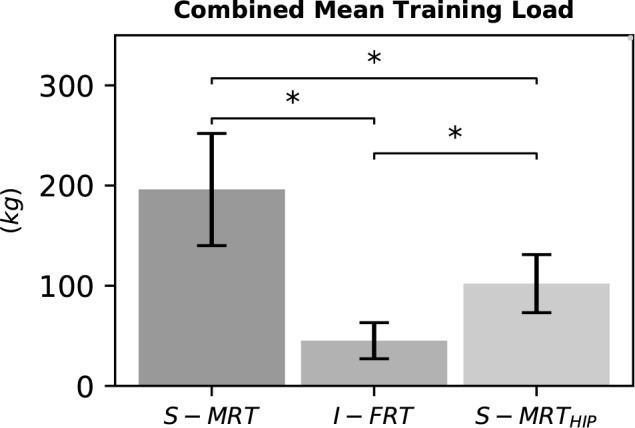
Combined mean load of the two main exercises during the last training phase. S-MRT, stable machine-based resistance training; I-FRT, instability free-weight resistance training; S-MRT_HIP_, stable machine-based adductor/abductor resistance training. Error bars represent standard deviation. Asterisk “^∗^”indicates a significant difference (*p* ≤ 0.05).

We found meaningful differences between groups, demonstrating that I-FRT exercised with considerably lower loads than the other groups. On average, I-FRT exercised on both main exercises with ∼150 kg less than S-MRT and with ∼56 kg less than S-MRT_HIP_. See Figure 6.

#### Questionnaire

Fear of falling, measured with the FES-I, was significantly reduced over time by 3–7% but the effects were similar across groups. M-SRT reduced the FES-I score from 19.3 ± 2.6 to 18.0 ± 2.3, I-FRT from 20.0 ± 3.7 to 18.4 ± 2.8, and S-MRT_HIP_ changed the score from 18.9 ± 3.2 to 18.3 ± 2.4.

## Discussion

The purpose of this study was to quantify the effect of different RT modalities on kinematic synergies (derived using UCM analysis) related to the ML trajectory of the swing-foot during normal and perturbed gait (walking across an uneven surface with and without additional weight/imbalance). Our first hypothesis, that the kinematic synergy for the ES condition would remain invariant for all groups after the intervention was supported. Our second hypothesis that the kinematic synergy during both unstable conditions would increase after training for the F-IRT group only was also supported. We found decisive evidence for an increased magnitude of the synergy index in the US and in the US_ISB_ condition. Consistent with our third hypothesis these stronger synergies were due to reduced “bad” variance for I-FRT while walking across the more challenging conditions; i.e., US and US_ISB_. All groups were able to rely on motor flexibility (proportional increase of “good” variability in relation to “bad” variability) to maintain a kinematic synergy at post-testing and across conditions. To the best of our knowledge, this is the first study investigating the use of the UCM approach to quantify (instability) resistance training induced changes within multi-segmental lower-extremity kinematic synergies to stabilize an important performance variable (i.e., ML swing-foot trajectory).

Previous research using the UCM analysis to investigate practice effects on coordination suggested a two-stage process of adaption in multi-segmental coordination (Wu and Latash, 2014). In the first stage “bad” variability drops due to optimized performance control, while “good” variability hardly changes. The second stage is characterized by a reduction in “good” variability, while “bad” variability remains constant. This can be explained by practice-induced optimized control over elemental features, other than the explicit performance variable (Wu and Latash, 2014). However, there are scenarios where a practice-induced increase of V_UCM_ can be found. An increase of V_UCM_ suggests a more robust and flexible system which can exploit an abundance of motor solutions, especially when being challenged (Wu and Latash, 2014; Latash and Huang, 2015). Our results certainly support the first stage, given the drop of V_ORT_ during both US conditions and the accompanying increase in the kinematic synergy. The fact that we observed an increase rather than a drop in V_UCM_ during the US conditions may be explained by increased gait speed. Given that gait speed increased following the intervention and that variability increases with speed, particularly at faster-than-preferred speed (Chien et al., 2015), we expected an increase particularly in performance destabilizing “bad” variability (Rosenblatt et al., 2014, 2015; Chien et al., 2015). With regard to the specific variance components, increases in movement speed have previously been associated with increased V_ORT_ (Chien et al., 2015; Rosenblatt et al., 2015) which is observed in the two machine-based groups. To counter the increased V_ORT_ these groups rely on motor flexibility and increase V_UCM_ as well, which is consistent with our previous cross-sectional study in which older adults compensated for challenged stability during walking by increasing V_UCM_ (Eckardt and Rosenblatt, 2018). The fact that I-FRT decreased V_ORT_ while walking across both US conditions despite increased gait speed is therefore noteworthy and consistent with the second stage of motor learning (Wu and Latash, 2014). The concurrent increase in V_UCM_ for this group highlights the fact that I-FRT specifically improved coordination, not to compensate for an increase in V_ORT_ but as means to develop coordination among elements to allow flexible performance and avoid reliance on a unique solution (Wu and Latash, 2014; Latash and Huang, 2015).

Several prior studies support the idea that resistance training incorporating modalities that promote exploration of movement space may positively affect motor flexibility. For example, Hamed et al. (2018) showed that while both I-FRT (or exercise dynamic stability under unstable conditions as they call it) and S-MRT improved muscle strength and balance recovery in simulated forward falls, only I-FRT increased standing balance abilities. The authors attributed the effects of I-FRT on the fact that the RT is performed under continuously destabilizing conditions that require continuous processing and integration of sensory afferent information to attain an appropriate motor response (Hamed et al., 2018), which is critical to control of dynamic postural stability under the unstable conditions. Exercising under unpredictable/instable conditions requires motor output to remain flexible enough to produce appropriate responses to continuously changing input. Indeed, recent investigations on adaptive mechanisms during uneven surface walking and running shows a widening of neuromuscular synergies (EMG) to provide robust and flexible motor solutions to compensate for perturbations (Santuz et al., 2018). Interventions forcing the CNS to explore an abundant number of motor solutions can elicit robust and more flexible neuromuscular synergies (EMG) (Oliveira et al., 2017).

In addition to increasing kinematic synergies, we found that I-FRT resulted in an increase in leg extension strength, which is in contrast to a previous RCT by our group (Eckardt, 2016). Indeed, the improvements for I-FRT in this study are larger than the prior RCT (Cohen’s *d*: 0.50 vs. 0.65). A different protocol to estimate training load may explain this difference. In our prior study, the 1-RM was calculated on the respective instability device whereas the current study calculated the training load on even surface. However, it may be difficult to elicit a true 1-RM on instability devices. In fact, the 1-RM for I-FRT was slightly higher in this study compared to the prior one (8 ± 4 kg) which may elicit the present effect and the subsequent interaction effect *time x group*.

It was somewhat unexpected that S-MRT_HIP_ did not improve adductor/abductor strength, which would be predicted based on principles of specificity (Buckthorpe et al., 2015; Contreras et al., 2016). We assume that the hand-held dynamometry test hip strength did not isolate hip adductors/abductors such that the other groups (i.e., S-MRT and I-FRT) could have attained similar testing values by compensating for weaker adductor/abductors with activation of other muscles to coordinate hip movement. Regardless of the reason, the fact that hip adductor/abductor strength did not improve with S-MRT_HIP_, or any of the other modalities, but that changes in the kinematic synergy were seen in I-FRT suggests that the neuromuscular strategies contributing to mediolateral foot placement and in turn synergistic behavior of lower-extremities may depend more on sensorimotor integration during dynamic (i.e., instable) situations than on force producing capabilities of the hip. Indeed both, S-MRT and S-MRT_HIP_ are quite stationary modalities, at least with regarding to the resistance components which were the primary training components; during resistive exercise execution there is little or no unpredicted movement and minimal counter-rotation of segments relative to the CoM (Hamed et al., 2018). Future work should consider individual effects – e.g., are kinematic synergies different between responders (those who increase adductor/adductor strength) and non-responders or do kinematic synergies scale with strength – to better understand the relationship between hip strength and kinematic synergies during gait.

## Limitations

Because our participants were healthy older adults (mean TUG time at pre-testing: 8.1 s), generalizability to frail older people needs to be established. In addition, the extent to which I-FRT can strengthen synergies (kinematic or otherwise) related to other performance variables has yet to be determined.

Given that the I-FRT group practiced walking across the US, it is not possible to entirely disentangle the impact of repeated US walking from instability RT on kinematic synergies. However, given that S-MRT practiced walking with weights and S-MRT_HIP_ practiced resisted walking yet demonstrated an increase rather than a reduction in V_ORT_, it appears that RT training alone, in absence of instability, cannot explain the changes observed in the I-FRT group. In addition, the fact that we observed similar strength changes but differences in motor flexibility between I-FRT and S-MRT suggests that the context in which strength changes occur is critical to promoting motor flexibility.

The high inter-subject variability (see Figure 4, 5) may indicate that there are individual strategies to stabilize the kinematic synergy. Future research should try to identify such subject specific strategies.

## Conclusion

The purpose of this RCT was to quantify how different resistance training modalities affect kinematic synergies related to the mediolateral trajectory of the swing-foot during normal and perturbed gait. For all groups the kinematic synergy during normal gait (ES condition) was unaffected by RT. However, I-FRT demonstrated significant increases in the kinematic synergy on the uneven surface which was achieved by reducing motor noise (V_ORT_) and therefore stabilizing the ML trajectory of the swing foot. To our knowledge, this is the first time the UCM approach was used to quantify resistance training induced effects on locomotor stability.

## Data Availability

All datasets analyzed for this study are included in the Supplementary Material SI.

## Author Contributions

NE analyzed the data, wrote the manuscript, and contributed to the conception and design of the study. NR contributed to the study design, assisted with the data analysis and interpretation, and critical review of the manuscripts. Both authors read and approved the final version of the manuscript.

## Conflict of Interest Statement

The authors declare that the research was conducted in the absence of any commercial or financial relationships that could be construed as a potential conflict of interest.

## Supplementary Material

MATERIAL SI Dataset.

MATERIAL SII Detailed UCM approach.
