# Supplementary material for: Instability Resistance Training Decreases Motor Noise During Challenging Walking Tasks in Older Adults: A 10-Week Double-Blinded RCT
Source: Front Aging Neurosci. 2019 Feb 27;11:32. doi: 10.3389/fnagi.2019.00032 (PMC6400996; doi:10.3389/fnagi.2019.00032)
Supplement: MATERIAL SII — Detailed UCM approach. [file Presentation_1.pdf]

Supplementary Material II

Instability resistance training decreases motor noise during challenging walking tasks in older adults: a 10-week double-blinded RCT

Nils Eckardt[1,2] & Noah J. Rosenblatt[3]

[1]Department of Training and Movement Science, Institute for Sport and Sports Science, University of Kassel, Germany

[2]Department of Sport and Movement Science, Institute of Sport Science, Carl von Ossietzky University of Oldenburg, Germany

[3]Dr. William M. Scholl College of Podiatric Medicine's Center for Lower Extremity Ambulatory Research (CLEAR), Rosalind Franklin University of Medicine and Science, North Chicago, IL, USA

*** Correspondence:**
Nils Eckardt
nils.eckardtl@uol.de

1 Detailed description of the uncontrolled manifold approach

First, we created a four-segment and seven degrees of freedom (DoF) geometrical model from which we derived an analytical expression for the trajectory of the swing limb ankle joint center relative to the stance limb (AJC_{GM}), i.e. our performance variable, based on the elemental variables, i.e. the seven DoFs (Θ). In light of the role of the stance limb mechanics in predicting upcoming foot placement it is quite reasonable that his performance variable would represent an important measure regarding mechanical stability of gait. The four segments of the model were: a stance limb (L_1), pelvis (L_2), swing-limb thigh (L_3), and swing-limb shank (L_4). The seven DoF of the model included the angles of each of the segments relative to vertical in the frontal plane (4 DoFs) and 3 DoF that relate to the motion of segments $L_{1\text{-}3}$ outside of the frontal plane and account for changes in the effective length of the segments when projected onto the frontal plane (Fig. 2). The hip joint centers were calculated from the experimental data according to the CODA pelvis (Bell et al., 1989), which is standard procedure for the IOR model. The ankle and knee joint centers were computed by using the distal end of the shank and thigh segment, respectively, as calculated from the experimental data. Accordingly, the geometric-model derived trajectory of the ankle joint and the elemental variable matrix were defined

$$\mathrm{AJC_{GM}} = L_1 \cos\alpha \sin\theta_1 + L_2 \cos\beta \cos\theta_2 + L_3 \cos\gamma \sin\theta_3 + L_4 \cos\gamma \sin\theta_4 \qquad (1)$$

$$\Theta = [\theta_1\ \theta_2\ \theta_3\ \theta_4\ \alpha\ \beta\ \gamma] \quad (2)$$

The experimental data was then used to drive the geometric model. First, elemental variables and segment lengths were derived from the three-dimensional experimental data then time normalized from toe-off until footstrike of the swing-foot (0 - 100%). For normalization, gait events were identified using a velocity based algorithm (O'Connor et al., 2007), which has been validated for use on uneven surfaces (Eckardt and Kibele, 2017). Next, to minimize the error between AJC_{GM} and the experimental trajectory of the swing-foot ankle joint center relative to the stance foot ankle joint center (AJC_{exp}), a subject specific optimization process was employed. For optimization, segment lengths were simultaneously varied from 90-110% of their original values and the set of values that minimized the error (AJC_{exp} - AJC_{GM}) were noted. AJC_{GM}, was then calculated at every percent of swing based on the optimized lengths. Finally, steps were removed from further analysis if either: 1) more than 25% of the normalized AJC_{GM} signal fell outside of the 95% confidence intervals for AJC_{exp}; or 2) the cross correlation between AJC_{GM} and AJC_{exp} was < .70.

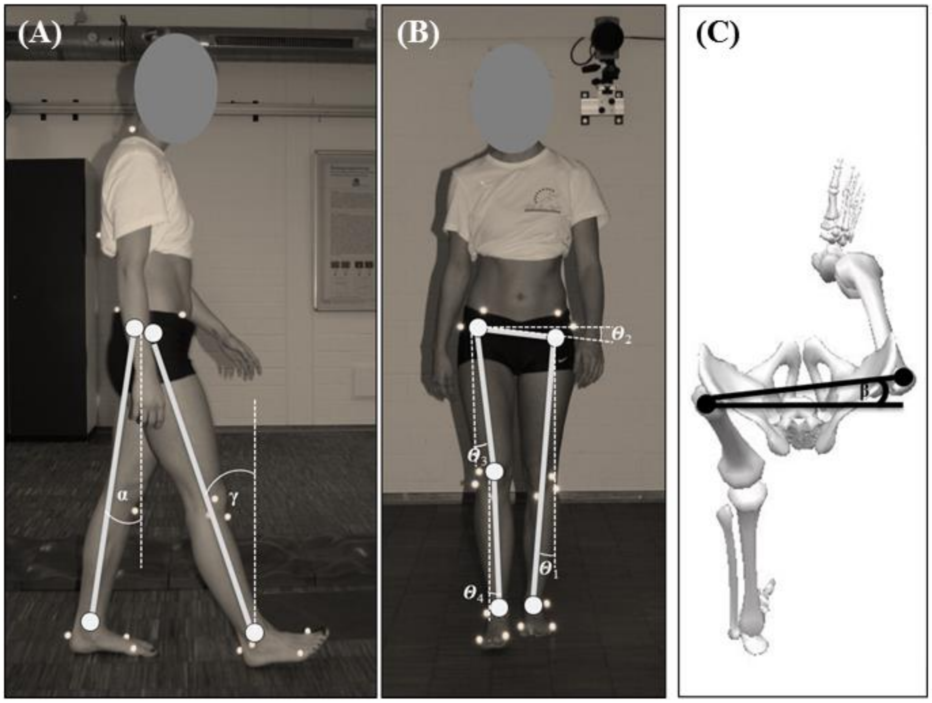

FIGURE 1 | The geometric model used to derive the analytical expression. (A) represents the sagittal plane view; **(B)** the frontal plane view and **(C)** the transverse plane view. In these figures right is the swing-leg and left the stance leg.

We derived the Jacobian matrix (*J*) from the forward kinematic model, to express how changes in the elemental variables affect the output of the geometric model

$$\mathrm{J} = \left[\frac{\partial \mathrm{AJC_{GM}}}{\partial \Theta}\right] = \; [L_1 \cos\alpha \cos\theta_1 \,, -L_2 \cos\beta \sin\theta_2 \,, L_3 \cos\gamma \cos\theta_3 \,, L_4 \operatorname{cosr} \; \gamma \cos\theta_4, \\ -L_1 \sin\alpha \sin\theta_1 \,, -L_2 \; \sin\beta \cos\theta_2 \,, \qquad -L_3 \sin\gamma \sin\theta_3 \,, L_4 \; \sin\gamma \sin\theta_4 \tag{3}$$

At every percent of swing we calculated the average value of the elemental variable matrix $(\overline{\Theta})$ and the Jacobian was then evaluated around $\overline{\Theta}$ to create a linearized approximation of the forward kinematics:

$$\mathrm{AJC_{lin}} = \overline{\mathrm{AJC_{GM}}} + \mathrm{J}(\overline{\Theta})(\Theta - \overline{\Theta}) \tag{4}$$

where $\overline{\mathrm{AJC_{GM}}}$ is the average value of the geometric model solution at each percent of swing. For all subjects at all points of the analysis (0-100% of the swing-phase) we verified that:

$$\mathrm{AJC_{exp}} - \mathrm{AJC_{lin}} << \; \mathrm{AJC_{SD}} \tag{5}$$

where $\mathrm{AJC_{SD}}$ is the standard deviation of the experimental swing-foot trajectory across all included steps. This was done to ensure that any error between the experimental data and linearized expression would have limited influence on the ensuing analysis.

After constricting the Jacobian, we used matrix decomposition (QR-decomposition) to compute the null-space of the Jacobian (ε):

$$\left(\mathrm{J}(\overline{\Theta})\right) \varepsilon = 0 \tag{6}$$

This null-space represents the uncontrolled manifold (UCM), which defines all combinations of Θ that do not cause the linearized solution, and by analogy $\mathrm{AJC_{exp}}$, to vary form average behavior. For

the *n* = 7 dimensional Θ and *d* = 1 dimensional AJC_{exp}, there exist *n-d* basis vectors *ε* that span the UCM. At every percentage of swing, the deviations of the segmental configurations from their means $(\Theta - \overline{\Theta})$ were projected onto the null-space:

$$\Theta_{\mathrm{UCM}} = \sum_{i=1}^{n-d} (\varepsilon_i \, (\Theta - \overline{\Theta})) \, \varepsilon_i \tag{7}$$

and onto a component orthogonal to this subspace:

$$\Theta_{\mathrm{ORT}} = (\Theta - \overline{\Theta}) - \Theta_{\mathrm{UCM}} \tag{8}$$

The variance in Θ that did not affect $AJC_{exp,}$ per DOF in the UCM ("good" variance) was calculated as the average squared length of Θ_{UCM} per DoF over all *N* steps:

$$\mathrm{V_{UCM}} = \frac{1}{(N)} \frac{1}{n-d} \sum_{i=1}^{N} (\Theta_{\mathrm{UCM}_i}^2) \tag{9}$$

Similarly, the variance in Θ that did affect AJC_{exp} ("bad" variance), per DOF in the orthogonal subspace was calculated as:

$$\mathrm{V_{ORT}} = \frac{1}{(N)} \frac{1}{d} \sum_{i=1}^{N} (\Theta_{ORT_i}^2) \tag{10}$$

Additionally, the total variance in segment configuration space per total DOF was determined as:

$$V_{TOT} = \left(\frac{1}{n}\right)(dV_{ORT} + (n-d)V_{UCM}) \tag{11}$$

If, statistically, $V_{UCM} > V_{ORT}$ then a lower-limb kinematic synergy exists to stabilize the mediolateral trajectory of the swing-foot (i.e., the elemental variables co-vary so as to reduce the variance of the mediolateral trajectory of the swing-foot). The strength of the synergy is defined by a synergy index:

$$\Delta V = \frac{V_{UCM} - V_{ORT}}{V_{TOT}} \tag{12}$$

For the current model, ΔV ranges from −7 (all variance is partitioned into V_{ORT}) to 7/6 (all variance is partitioned into V_{UCM}). For statistical analysis, consistent with previous studies (Rosenblatt et al., 2015; Verrel, 2010) we used a modified Fischer's z-transformation to normalize the synergy index (Solnik et al., 2013) prior to statistical analysis:

$$\Delta Vz = \frac{1}{2}\ln\left[\frac{(7+\Delta V)}{\left(\frac{7}{6} - \Delta V\right)}\right] \tag{13}$$

The existence of a synergy ($\Delta V > 0$) corresponds with $\Delta Vz > 0.89$. The complete R-code we applied is available from the corresponding author (NE) upon request. For each subject we averaged the variance components and z-transformed synergy index across the gait cycle before entering the data into statistical analysis.

1.1 References

Bell, A. L., Brand, R. A., and Pedersen, D. R. (1989). Prediction of hip joint centre location from external landmarks. *Hum. Mov. Sci.* 8, 3–16. doi:10.1016/0167-9457(89)90020-1.

Eckardt, N., and Kibele, A. (2017). Automatic identification of gait events during walking on uneven surfaces. *Gait Posture* 52, 83–86. doi:10.1016/j.gaitpost.2016.11.029.

O'Connor, C. M., Thorpe, S. K., O'Malley, M. J., and Vaughan, C. L. (2007). Automatic detection of gait events using kinematic data. *Gait Posture* 25, 469–474. doi:10.1016/j.gaitpost.2006.05.016.

Rosenblatt, N. J., Latash, M. L., Hurt, C. P., and Grabiner, M. D. (2015). Challenging gait leads to stronger lower-limb kinematic synergies: The effects of walking within a more narrow pathway. *Neurosci. Lett.* 600, 110–114. doi:10.1016/j.neulet.2015.05.039.

Solnik, S., Pazin, N., Coelho, C. J., Rosenbaum, D. A., Scholz, J. P., Zatsiorsky, V. M., et al. (2013). End-state comfort and joint configuration variance during reaching. *Exp. Brain Res.* 225, 431–442. doi:10.1007/s00221-012-3383-2.

Verrel, J. (2010). Distributional properties and variance-stabilizing transformations for measures of uncontrolled manifold effects. *J. Neurosci. Methods* 191, 166–170. doi:10.1016/j.jneumeth.2010.06.016.
